# Rule reactivation and capture errors in goal directed behaviour

**DOI:** 10.1016/j.cortex.2017.08.027

**Published:** 2018-10

**Authors:** María Roca, Milagros García, María Juliana Torres Ardila, María Luz González Gadea, Teresa Torralva, Jesica Ferrari, Agustín Ibáñez, Facundo Manes, John Duncan

**Affiliations:** a Institute of Cognitive and Translational Neuroscience (INCyT), INECO Foundation, Favaloro University, Buenos Aires, Argentina; b National Scientific and Technical Research Council (CONICET), Buenos Aires, Argentina; c Center for Social and Cognitive Neuroscience (CSCN), School of Psychology, Universidad Adolfo Ibáñez, Santiago de Chile, Chile; d Centre of Excellence in Cognition and Its Disorders, Australian Research Council (ACR), Sydney, Australia; e Universidad Autónoma del Caribe, Barranquilla, Atlántico, Colombia; f MRC Cognition and Brain Sciences Unit, Cambridge, UK

**Keywords:** Capture errors, Fluid intelligence, Rule interference, Goal neglect

## Abstract

In everyday life people may act automatically, following “unwanted” lines of action which are triggered by contextual cues and may interfere with current goals. Such occurrences are known as “capture errors” in reference to errors that occur when a more salient behaviour takes place when a similar, but less salient, action was intended. Clinical neuropsychological studies suggest that reactivation of previous rules may play an important role in behavioural interference, but such reactivation has been little studied in normal subjects and simple experimental tasks. In the present study we develop this theme, presenting data on 4 subjects who spontaneously showed capture errors in verbal fluency tasks, and developing a new experimental paradigm specifically designed to elicit such interference in normal subjects. In the new paradigm, 101 normal subjects performed a simple series of working memory tasks, including occasional stimuli whose answer matched both the current and the previous rule. We found that normal controls indeed tend to commit more mistakes after the presentation of a stimulus whose answer is consistent with a current and preceding rule. In this case, however, the errors produced are not necessarily associated with a shift back to the old rule, suggesting that rule reactivation leads to a more general interference effect. We discuss the importance of our data from both theoretical and clinical perspectives.

## 1 Introduction

In many everyday situations, people act disregarding their explicitly intended goals, automatically following other lines of actions that were not part of their original plan. An example of this not infrequent situation could be the following: one morning you get up with the clear intention to do something, for instance to electronically pay your bills. When you turn on your computer and click the browser icon, your emails get in your way. Your original idea fizzles and another goal takes charge. You start reading and replying to emails, including some which are clearly much less important than having the power cut off due to lack of payment (which, by the way, will end any further possibility of reading your emails). This “unwanted” behaviour seems to be automatically triggered by cues in the context that drag us to other lines of action interfering with our original aims and goals. In the given example, the sole glance at the browser icon is enough to make you disregard your original plan. This replacement of some goals with others happens in countless situations of everyday life and most of us live with it, without further consequences.

In human psychology, such occurrences have been described as “slips” or “capture errors”, referring to errors that occur when a more salient or practised behaviour takes place when a similar, but less salient, action was intended (Norman, 1981, Norman, 1983, Reason, 1990). This term has been widely used in different fields including client usability, and in both medical and legal psychology. In one case, for example, two police officers claimed to have shot a suspect while supposedly intending to use their taser (Shoichet, Morris, & Lavandera, 2015). Even if the importance of such mistakes has long been recognized, there is not much research performed to elucidate which variables – regarding the subject and the action context – predispose to this kind of error in normal subjects.

Patients with frontal lobe pathology show slips and capture errors at multiple levels. In anarchic hand, for example, objects in the environment may draw out compulsive, unwanted actions from the affected hand (Della Sala et al., 1991, Riddoch et al., 1998). In action disorganization syndrome, familiar sequences of behaviour are interrupted by frequent intrusions, including re-insertions of a step already completed (Humphreys and Forde, 1998, Schwartz et al., 1991). In “goal neglect” (Duncan, Emslie, Williams, Johnson, & Freer, 1996), behaviour is captured by the wrong element of a novel task, even though the patient clearly understands and states what behaviour is required. In the Duncan et al. (1996) study, participants were asked to perform a simple monitoring task involving pairs of letters and numbers. Subjects were directed by a main cue (“WATCH LEFT” or “WATCH RIGHT”) and were asked to name the letters appearing on the side indicated by the cue while ignoring digits. Near the end of each trial, a second, more abstract cue (a plus or minus symbol) appeared and directed participants to two possible lines of action: if a minus appeared then subjects should start naming the letters on the left, no matter which side they had previously been watching, and if a plus appeared, the subject should name letters from the right. On some trials the symbol meant that the subject should continue to answer as they had been doing earlier (WATCH RIGHT followed by a +; WATCH LEFT followed by a −) and on other trials the sign meant that they had to shift their response (WATCH RIGHT followed by a −; WATCH LEFT followed by a +). Duncan et al. (1996) found that some participants tended to neglect the second and more abstract rule even if they could recall it at the beginning and at the end of the task. Even though they knew they should respond to the cue, they systematically ignored it, appearing “captured” by the initial part of the task. After this paper, similar behaviour has been repeatedly reported using different research paradigms and it has been described in children, older adults and multiple clinical populations (Bhandari and Duncan, 2014, Duncan et al., 2008, Duncan et al., 2012, Nieuwenhuis et al., 2004, Roca et al., 2014, Towse et al., 2007, Westbrook et al., 2012).

Certain subject characteristics such as age, fluid intelligence, and working memory capacity seem to be related to goal neglect behaviour. Children and older adults seem to have a tendency to present this behaviour (Nieuwenhuis et al., 2004, Towse et al., 2007, Westbrook et al., 2012), as do people with lower scores on fluid intelligence tests (Bhandari and Duncan, 2014, Duncan et al., 1996, Duncan et al., 2008, Kane and Engle, 2002) or poor working memory capacity (Kane and Engle, 2002, Oberauer, 2010).

Though some task characteristics are known to influence goal neglect – such as task complexity and progress within a task (Bhandari and Duncan, 2014, Duncan et al., 2008, Duncan et al., 2012, Kane and Engle, 2002) – much less is known about how particular stimuli can provoke this brain stumble. Here we take our lead from a study of capture errors in frontal patients reported by Reverberi, Lavaroni, Gigli, Skrap, and Shallice (2005). These authors used a modified form of the well-known Brixton spatial anticipation task (Burgess & Shallice, 1996) in which participants are presented with a series of cards containing a display of circles one of which is blue. The main goal of the subject is to anticipate which circle will be the blue one in the following card, by inferring the rule from previous cards. The Reverberi version of the task (2005) adds an interesting rule interference component. Once the rule is inferred, 4 cards with a red circle, following a different rule, appear. The subject is explicitly told that in those cards he should only touch the red circle, and that those cards are irrelevant to the main task. The subject is also told that after the red cards the blue circle will always continue to follow the same rule as before the interruption and that this preceding rule is the one the subject has to follow once the series of red circles finishes. Interestingly, the succession of the interfering red cards is arranged so that the position of the next blue card fits the rule of both red and blue circles. Under these circumstances, Reverberi et al. (2005) found that, when the next blue card was presented, some frontal patients followed the interfering (red card) rule, rather reverting to the correct (blue card) rule. Such results suggest that the conjoint activation of two rules may play an important role in rule interference. In this study, however, the first blue card following a series of reds always fit both rules, making it uncertain whether this double match was crucial in generating capture errors. To date, furthermore, there are no studies exploring similar capture in normal subjects.

In clinical neuropsychology, a similar kind of mistake is also sometimes observed in simpler tasks, such as verbal fluency. In verbal fluency tasks, subjects are asked to generate as many items as possible from a given category in a fixed time. Classically, a phonemic and a semantic version are used. In the first, the subject is asked to generate words beginning with a given letter – for example “f” – while in the second, the subject is asked to generate words from a given category – for example animals or types of food. Interestingly, when the two tests are presented in succession, it is not rare to find something like the “capture errors” previously described. That is, the old rule – for example generating words with the letter “f” – is re-activated in the semantic fluency task, if by chance an answer coherent with both rules is produced. For example, during the animal fluency task, if the subject arbitrarily says the word “fox”, then the old rule may interfere and he/she starts generating animals exclusively starting with the letter “f” or, in a more severe case, may even revert to the old rule, generating words beginning with “f” whether or not they match the required semantic category.

In the present study we develop this theme of interference arising when a response is coherent both with a current task rule and a previous, now irrelevant rule. First, we present data on 4 subjects who spontaneously showed this kind of behaviour in verbal fluency tasks. Then, we present data from an experimental paradigm specifically designed to elicit such interference. In this study 101 normal subjects performed a simple series of neuropsychological tasks, including occasional stimuli whose answer matched both the current and the previous rule. We examine interference induced by such double matches, and relate it to a number of task characteristics.

## 2 Cases

In this section we present data on 4 subjects who spontaneously showed capture behaviour in verbal fluency tasks. The 4 cases were retrospectively identified from a group of 52 subjects that were part of an ongoing investigation on Alzheimer's Disease and Fronto-temporal Dementia (FTD). Of the subjects reviewed, 10 were cases of progressive primary aphasia, 16 cases of behavioural variant FTD, 9 cases of Alzheimer's disease and 17 healthy controls. As part of the study, patients were assessed with a complete neuropsychological battery that included a phonological verbal fluency task immediately followed by a categorical verbal fluency task. First, the subject was asked to produce as many words as possible beginning with the letter “p” in 1 min. After this task was finished, subjects were asked to produce as many animals as possible in 1 min. Subjects were explicitly told that they could mention any animal, disregarding the initial letter.

### 2.1 Case 1

FM was a right-handed 70-year-old male who initially presented with behavioural disorders and attentional deficits. His family reported apathy, irritability and appetite augmentation. No memory, language, visuoperceptual or orientation deficits were initially reported. Neuropsychological assessment showed difficulties in executive functions, theory of mind and multitasking, while memory, language and praxis were preserved. Brain MRI indicated moderate bilateral frontal involution. FM received a diagnosis of the behavioural variant of FTD and was invited to take part in the study. When presented with the phonological verbal fluency task, he produced 6 words in the allowed 1 min. Afterwards, when presented with the categorical verbal fluency task, after correctly mentioning 4 animals, he produced an animal whose initial letter was the letter “p”. Immediately after, he switched to the former task and started to produce words with the letter “p” that were not animals till the task was finished. Of note, the first animal produced also started with the letter “p”.

### 2.2 Case 2

DF was a right-handed 64-year-old male IT technician. When he was 61 years old he presented progressive behavioural changes and emotional liability. Within 2 years his deficits progressed to other cognitive functions including memory and language, with word finding difficulties. Behavioural deficits became more prominent including hyperorality and marked inappropriate behaviours. MRI indicated clear bilateral frontotemporal involution with left frontal predominance. DF received a diagnosis of behavioural variant of FTD and was invited to take part in the study. When presented with the phonological verbal fluency task, he produced 7 words in the allowed 1 min. When subsequently he was asked to generate animals, he produced 3 in the first 15 sec, with the first one starting with the letter “p”. Around second 15 he produced a new animal beginning with the letter “p” and then switched back to the former task, producing a word with the letter “p” that was not an animal.

### 2.3 Case 3

DG was a right-handed 79 year old woman who complained of widespread musculoskeletal pain and fatigue, within many other physical symptoms. Given other transient symptoms such as dizziness, tension headache, tinnitus, cold feet, dry mouth and difficulty to swallow, she was seen by doctors from different specialities. She did not present pathological findings during her neurological examination nor any other explanation for her symptoms. An informant reported other changes of behaviour including cognitive rigidity and perseverative behaviours and thoughts. Her brain SPECT showed hypoperfusion in bilateral anterior and medial frontal lobes. When she was presented with the phonological fluency task, she produced 16 words in the given minute. Subsequently, in the categorical fluency task around second 15, she produced an animal which started with the letter “p”. Immediately afterwards she produced a word starting with the letter “p” that was not an animal.

### 2.4 Case 4

MH was a right-handed 70-year-old female lawyer who volunteered to participate in the study as a control subject. She reported no cognitive or behavioural problems. When she was presented with the phonological verbal fluency task, she produced 15 words in the allowed 1 min. Subsequently, when she was asked to generate animals, she produced 8 in the first 30 sec. Around second 30 she generated an animal beginning with the letter “p” and then reverted to producing “p” words that were not animals.

## 3 Materials and methods

### 3.1 Participants

A sample of 101 healthy subjects was included in the study. Subjects were selected by the absence of prior neurological background, substance abuse or psychiatric antecedents. The mean age of subjects was 51 years (*SD* = 22.1; *range* = 18–90) and mean education level was 13.72 years (*SD* = 4.96; *range* = 3–30). Subjects were recruited by word of mouth and were not taking any medications indicating medical problems at the time of assessment. Subjects were evaluated individually in a suitable examination room. Permission for the study was obtained from the local research ethics committee in accordance with the 1964 Declaration of Helsinki and all subjects gave their informed consent.

### 3.2 Capture error task

The study used 4 simple tasks, involving verbal working memory for lists of numbers or numbers and letters. In each trial, a list was read aloud by the experimenter one digit or letter per second. A simple rule determined the correct order of repeating it back, with 4 different rules for the 4 different tasks. The first task was only included to consolidate the first rule and was not included in the statistical analysis. Each task consisted of 30 trials, and from the second to the fourth, it included 3 different types of stimulus: A) Trigger stimulus (TRIG; 6/30) which were lists for which the answer was the same for the current task and the immediately preceding one, in order to re-activate the previous and now irrelevant rule; B) Stumble stimulus (STMB; 6/30) which were the trials immediately following a TRIG, predicted to show more errors given the reactivation of the previous rule on the TRIG trial; and C) Regular stimulus (REG) which were all the remainder of the trials. TRIG stimuli were randomly distributed across the 30 trials of each task, with the exception of the first stimulus, which was always a TRIG in order to maximise the probability of reawakening the previous rule.

To manipulate the influence of working memory load (WML), the experiment was divided into two halves. The first half of the experiment used two item lists, with the 4 tasks performed in turn (30 trials/task). Then in the second half of the experiment, the whole cycle of 4 tasks was repeated with four item lists. The whole experiment took an average of 40 min to be completed.

In each half of the experiment, tasks were given in the following order. The complete list of stimuli for all tasks is shown in Supplementary Materials.

*Digit Forward Repetition (DF)*: The subject was presented with numbers and was asked to repeat them in the same order as presented. No TRIG stimuli were included.

*Digit Backwards Repetition (DB)*: In the second task, subjects were presented with numbers and were asked to repeat them in the reverse order from that in which they were presented. For this task, TRIGs consisted of stimuli for which the correct answer is the same forwards and backwards, meeting criteria for the former and current rule at the same time (example: 2-8-8-2).

*Letter and Number Organization (LNO)*: Subjects were now presented with numbers and letters, and were asked to repeat them in the order: first numbers in ascending order; then letters in alphabetical order. This task was included in order to investigate the effect of rule complexity, since it involves the same number of working memory items as DB but applying a more complex rule. For this task, TRIG stimuli were composed of letters and numbers presented in such a way that saying them backwards would produce the same answer assaying them following the LNO rule (for example: L – A – 4 – 1).

*Letters and Numbers Backward Repetition (LNB)*: Subjects were presented with numbers and letters and were asked to repeat them in the reverse order from that in which they were presented. This task was included to investigate the effect of rule complexity versus stimulus complexity, since it equals the rule complexity of DB but the stimulus complexity of LNO. For this task, TRIG stimuli were composed of letters and numbers presented in such a way that saying them following the letter and number organization rule would produce the same answer as said backwards (for example: L – A – 4 – 1).

### 3.3 Data analysis

For the statistical analysis, we measured the percentage of errors separately for TRIG, STMB and REG trials. We used repeated measures ANOVA in which we included as within subject measures: a) stimulus type (TRIG, STMB and REG), and b) task (DB, LNO and LNB). To examine the effect of WML, we used a separate repeated measures ANOVA including within subjects measures: a) stimulus type (TRIG, STMB and REG), b) task complexity (LNO and LNB), and c) WML (high and low). DB was removed from this analysis as the variance of the low WM condition was insufficient. We used a Tukey HSD test to contrast differences between conditions (*p* < .05). Pearson's correlations were calculated between performance and age, years of education and fluid intelligence measured by the matrix reasoning task (Wechsler, 1997).

## 4 Results

### 4.1 Type of stimulus effect

**Fig. 1:**
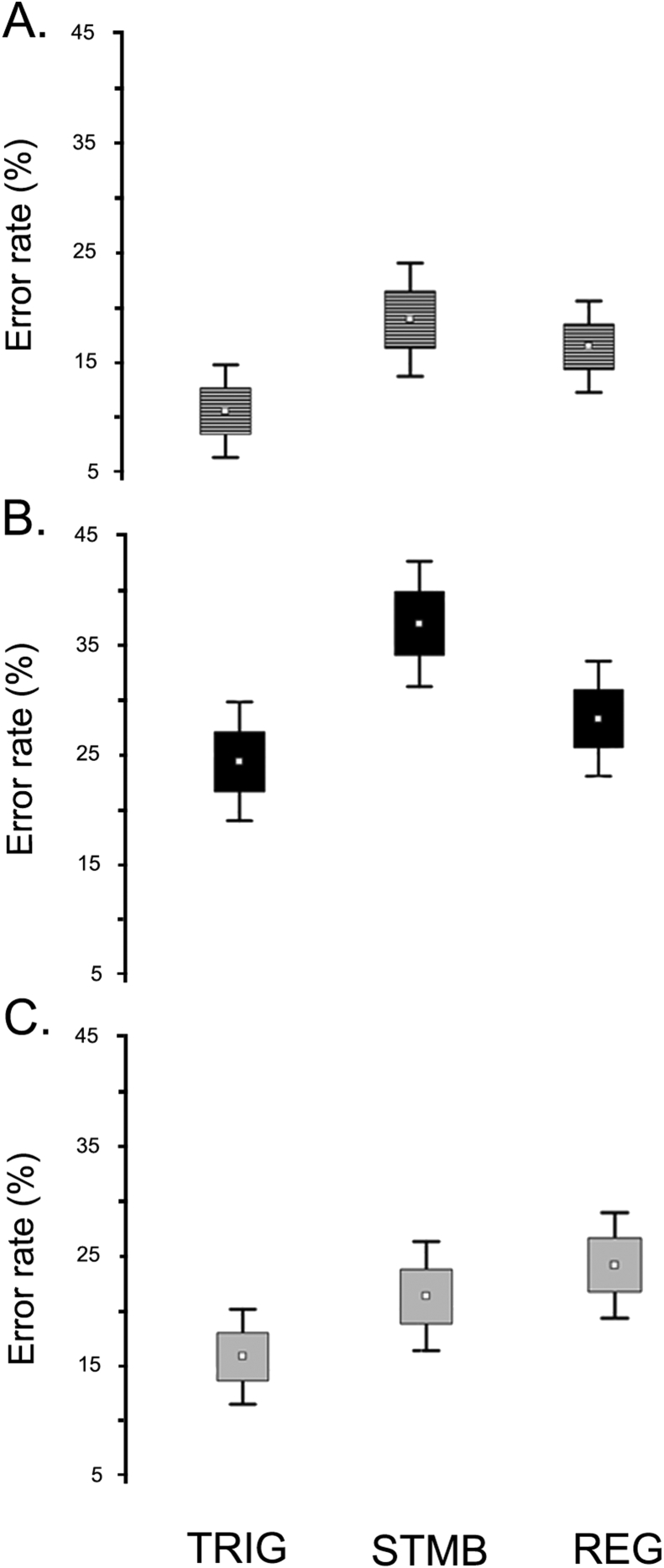
**Interaction between stimulus and task**. Box plots show the significant stimulus effect, where participants exhibited a higher percentage of errors on the STMB stimulus. The dot represents the mean, the box the mean ± SE, and the whiskers the mean ± .95 confidence intervals. **A**. Stimulus in the Digit Backwards Repetition (DB) task. **B**. Stimulus in the Letters and Numbers Backward Repetition (LNB) task. **C**. Stimulus in the Letter and Number Organization (LNO) task. TRIG: trigger stimulus; STMB: stumble stimulus; REG: regular stimulus.

Error percentages for each task and stimulus type are shown in Fig. 1. Data are means across the two levels of WML. To investigate the effects of a response consistent with the rules of both the current and the preceding task, we compared errors for TRIG, STMB and REG stimuli. As expected, we observed a significant effect of stimulus type [*F* (2,200) = 28.04; *p* < .001; *η*2 = .524]. A post hoc analysis (Tukey's HSD, MS = 214.30; df = 200.00), showed that participants performed significantly more poorly in STMB compared to both other stimulus types (error rate STMB > TRIG: *p* < .001; STMB > REG: *p* = .038). Accuracy was highest on TRIG trials (REG > TRIG: *p* < .001). Poor performance on STMB trials confirms the disturbing effect of TRIG stimuli in reawakening the previous rule. Reawakening of the previous rule could also have contributed to good performance on TRIG stimuli themselves, for which the reawakened rule was consistent with the correct response. It is also worth noting, however, that TRIG stimuli were relatively simple in the DB task, since they necessarily contained repeated numbers (e.g., 2-8-8-2).

### 4.2 Task effect

There was a significant effect of task type, *F* (2, 200) = 43.17; *p* < .001; *η*^2^ = .302. A post hoc analysis (Tukey's HSD, *MS* = 395.23; *df* = 200.00) showed that participants performed significantly more poorly in LNB compared to both other tasks (error rate LNB > LNO: *p* < .001; LNB > DB: *p* < .001). There was also a higher error rate in LNO compared to DB (*p* = .003) (see Fig. 1).

In addition, there was a significant interaction between stimulus type and task type, *F* (4,400) = 6.62; *p* < .001; *η*^2^ = .062. Post hoc tests (Tukey's HSD, *MS* = 150.34; *df* = 400.00) showed that in LNB task, error rate for STMB was higher than for either REG or TRIG (both *p* < .001). There was no significant difference between TRIG and REG stimuli (*p* = .470). In DB task, both STMB (*p* < .001) and REG (*p* = .023) showed higher error rate than TRIG. Last, in LNO, there was no difference between STMB and TRIG (*p* = .096), nor between STMB and REG (*p* = .670).

### 4.3 Working memory effect

**Fig. 2:**
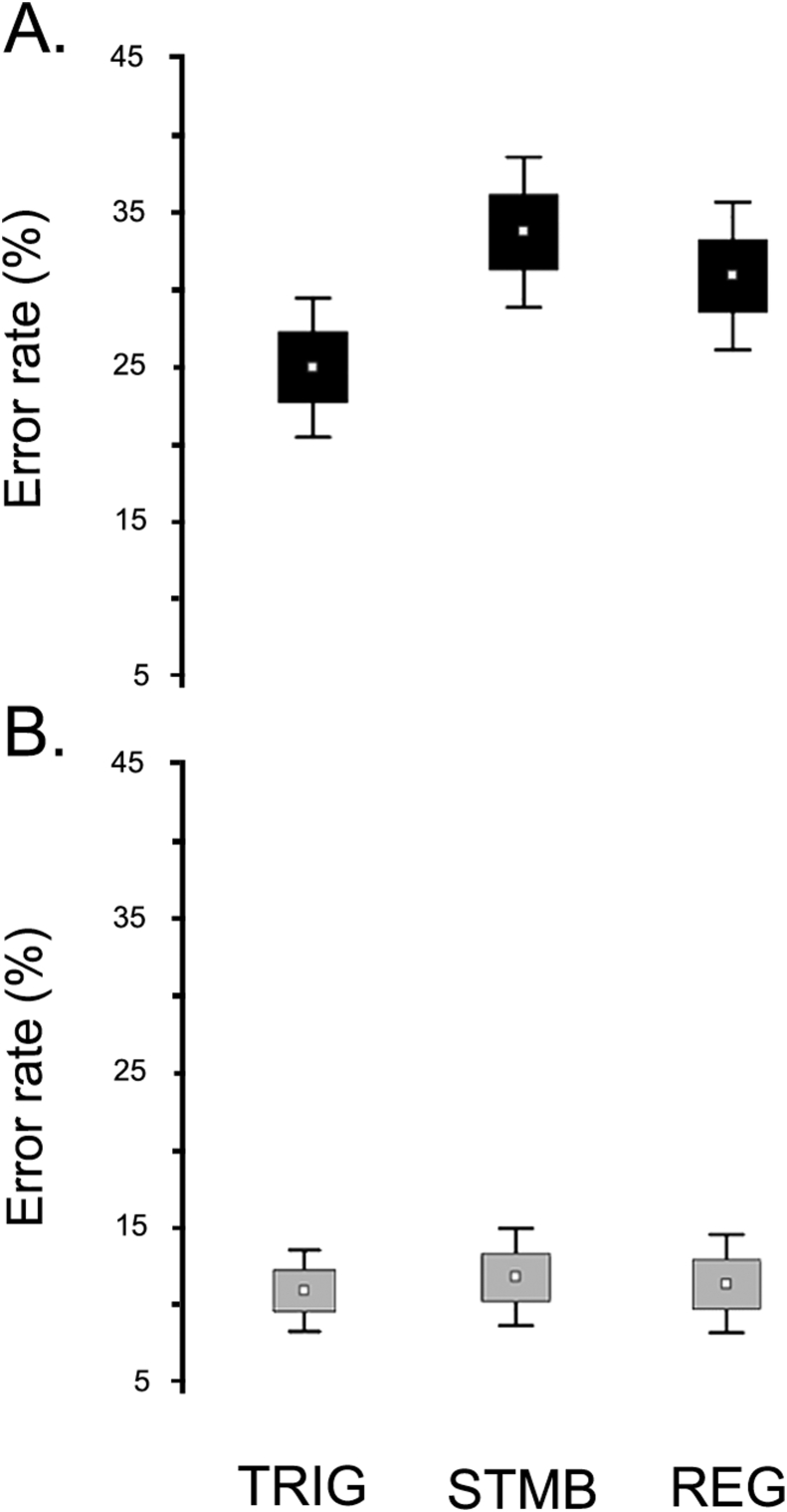
**Effect of working memory load**. Errors were significantly higher in the high WML condition than in the low WML condition. White dots represent the mean, the box the mean ± SE, and the whiskers the mean ± .95 confidence intervals. **A**. Stimulus in the high working memory load lists. **B**. Stimulus in the low working memory load conditions. TRIG: trigger stimulus; STMB: stumble stimulus; REG: regular stimulus.

As we expected, we found significant effects of WML [*F* (1,100) = 95,602; *p* < .001; *η*^2^ = .489], with lower performance during the high WML condition. Fig. 2 shows a ceiling effect for the Low WML condition, with high accuracy for all stimulus types.

We found a significant interaction between stimulus type and WML [*F* (2,200) = 10,257; *p* < .001; *η*^2^ = .165]. Post hoc analyses (Tukey's HSD, *MS* = 156.39; *df* = 200.00) revealed that TRIG stimuli significantly differed from both STMB (*p* < .001) and REG (*p* < .001) stimuli in the high WML condition. No significant differences between stimuli were observed in the Low WML condition.

### 4.4 Types of error

A final analysis concerned the nature of errors, in particular for four-item lists. For STMB and REG stimuli, the four items in a list were always all different (see Supplementary Materials), meaning that, if all items were reported, they could be in one of 24 possible orders. Of these, one order was correct, one was the order consistent with the preceding task (“back to old rule”, BOR), and the remaining 22 were other order errors (NoBOR). No similar analysis was possible for two-item lists, with only one possible order error, or for TRIG stimuli, where the BOR response was correct.

To see if TRIG stimuli specifically induced BOR errors, we performed repeated measures ANOVAs separately for each task, contrasting STMB, the trial that immediately followed a STMB (STMB+1), and all other REG stimuli (REG). BOR errors were very infrequent, and ANOVA showed no effect of stimulus type in the DB [*F* (2,200) = .284; *p* = .753; *η*^2^ = .003] and LNB tasks [*F* (2,200) = .459; *p* = .633; *η*^2^ = .918]. In the LNO task there were no BOR errors.

Regarding the number of NoBOR errors, we found significant differences in the LNO [*F* (2,200) = 17,144; *p* < .001; *η*^2^ = .146] and LNB tasks [*F* (2,200) = 14,589; *p* < .001; *η*^2^ = .127]. Post hoc analyses revealed that in the LNO task (Tukey's HSD, *MS* = 158.87; *df* = 200.00) NoBOR errors were higher for STMB+1 stimuli compared to STMB (*p* < .001) and REG (*p* < .001) stimulus. For LNB task (Tukey's HSD, *MS* = 83.43; *df* = 200.00) NoBOR errors were higher for STMB compared to STMB+1 (*p* = .004) and REG (*p* < .001). The DB task also showed no difference between stimulus types in the number of NoBOR errors (*F* (2,200) = 1408; *p* = .247; *η*^2^ = 2.816). These data show that, although a TRIG stimulus induced order errors in the following trial, in particular in LNO and LNB, these errors were not specifically responses consistent with the preceding task's rule.

## 5 Discussion

In the present study we examined a form of rule competition in sequential behaviour. Specifically we showed interference arising when a response generated according to a current task rule happens to be consistent with the rule of a preceding task.

To illustrate this, we first presented data on subjects drawn from an ongoing study of Alzheimer's disease and FTD. Of 52 cases retrospectively analysed, 4 showed a striking form of rule capture in verbal fluency tasks. All subjects performed a phonemic fluency task followed by a semantic fluency task. In each case, during the semantic fluency task, when a word beginning with the previously used letter happened to be generated, the subject reverted to the previous task, generating words that began with the letter used in the phonemic fluency task. Interestingly, of the 4 subjects showing this behaviour, 3 were suffering from the behavioural variant of FTD, while the fourth was a control subject. It seems likely that capture errors of this sort can be found in multiple populations, especially in patients with frontal dysfunction.

To further investigate this behaviour and its interaction with task and subject characteristics, we designed a new experimental paradigm. A large sample of normal subjects was presented with a simple series of working memory tasks, with rules changing from one task to the next. Occasionally, the correct answer for a stimulus list also matched the rule of the preceding task. When this occurred, error rate increased on the following trial. This effect was most visible in a task combining high stimulus complexity with a relatively simple response rule. It was also dependent on WML, being seen only in four-item but not two-item conditions.

Strikingly, when the types of errors produced were analysed, errors were not specifically responses consistent with the preceding task's rule. In this regard, unlike the data from verbal fluency, findings in our experimental task do not suggest a simple disappearance of the current rule and re-emergence of the previous one. Instead, our results suggest that the presentation of a stimulus whose answer is simultaneously compatible with a current and a previous rule generates interference with the task being performed and a general disturbance in use of the correct rule. This interference is reflected in an overall increase in order errors. At least for normal subjects, our data suggest that a TRIG stimulus is not enough to prompt a complete switch back to a previous mode of responding. We suggest that a stimulus that is congruent both with a current and a previous rule interferes with current rule application both in normal subjects and in clinical populations. Complete hijacking by a previous rule, however, may only be common in clinical populations. Testing this hypothesis will require testing of clinical and normal populations under the same conditions.

A further finding was reduction of error rate on TRIG trials, where the response required on the current trial was consistent both with the current rule, and with the rule of the preceding task. Although these results could suggest some remaining activation of a previous rule, the fact that better performance in TRIG stimuli was significant only in the DB condition suggests a role of stimulus complexity. In the DB condition, TRIG stimuli were unlike others in that they included repeated items (e.g., a TRIG stimulus in DB would be 2-2 or 2-4-4-2 while a REG or TMBL stimulus would be 5-2 or 7-3-1-9).

Here we have studied interference between the rules of two tasks performed in succession, in separate trial blocks. Our results have some similarities to findings in tasks like the Stroop or Eriksen flanker, which address interference between responses induced by different aspects of a current stimulus (e.g., Stroop, name ink colour, ignore written word). In tasks of this sort, it is common to compare congruent stimuli – for which relevant and irrelevant stimulus features indicate the same response (e.g., Stroop, word RED written in red ink) – with incongruent stimuli – for which the two features indicate different responses (e.g., Stroop, word RED written in green ink). In some respects, our TRIG stimuli resemble a Stroop or Eriksen congruent (both current and preceding rules lead to the same response), while other stimuli (STMB and REG) resemble a Stroop or Eriksen incongruent. In tasks like Stroop and Eriksen, reaction time on an incongruent trial is generally longer when the preceding trial was congruent (Gratton effect, see Gratton, Coles, & Donchin, 1992), precisely in line with our finding of increased errors for STMB, which follows a congruent TRIG. The most influential account of the Gratton effect proposes that, after an incongruent trial, suppression of the unwanted stimulus feature/task rule is strengthened, speeding performance if the next trial is also incongruent (Botvinick, Braver, Barch, Carter, & Cohen, 2001). Others have argued that, complementarily, a congruent may encourage processing of the irrelevant feature, impairing performance if the next trial is incongruent (e.g., Lamers & Roelofs, 2011). Though here we address a rather different type of interference, occurring between successive tasks over a longer time-scale, similar underlying control mechanisms may be at play.

The context in which daily activities take place is full of distractors, potentially interfering with the path to our goals. To avoid incorrect choices, we must precisely select actions which will lead us towards achieving our main goals. Countless examples of slips and capture errors can be found in everyday life, such as the described hijacking of your intention to pay your bills by your e-mails or when you end up accepting a call in your phone when you did not intended to do so. The present investigation illustrates the role of goal competition on such kind of mistakes both in clinical subjects and in healthy adults. We showed that in some cases the simple presentation of a response coherent with a previous rule can make some subjects shift back to it. We also showed that, in normal subjects, reactivation of a previous rule can interfere with simple tasks, even if there is no complete shift back to the previous rule. Such reactivation can interfere with goal management even in simple tasks with no rule induction components, and even with just a single triggering stimulus.

A woman gets up with the clear intention to electronically pay her bills but clicking the browser icon awakens the familiar process of checking her emails. Although he has previously refused to join the family football game, a professional footballer gets up and starts to play once the other parents “accidentally” kick the ball to his feet. This abduction of some goals over others happens in countless situations of everyday life, and sometimes, the consequences are serious (Norman, 1981, Norman, 1983, Reason, 1990). Accordingly, understanding and avoiding such mistakes is of great importance in multiple fields, from client usability in software programming to the design of medical practices and equipment.

Further investigations should be performed both in vulnerable populations – such as children, older subjects and patients with frontal dysfunction – and using different cognitive paradigms. This will allow us to better understand the strength and extent of this phenomenon. Such knowledge could be a starting point to design rehabilitation strategies focussed on stimulus interference control for goal management deficits observed in clinical populations.

## 6 Conclusions

The investigation of human error and the factors that can predispose to it is of great importance for different fields. In the present study we showed how the generation of an answer which is congruent both with a current and previous rule can disrupt performance. While in patients with frontotemporal dementia such mistakes seem to be related to a complete reversion to the previous rule, in normal subjects it seems to interfere more generally with performance, producing errors not necessarily associated with a shift back to the old rule.

## Declaration of interest

Authors declare no conflict of interest including any financial that could inappropriately influence their work.

## Funding

This work was supported by Medical Research Council (UK) intramural program [grant number MC-A060-5PQ10], CONICYT/FONDECYT Regular [grant number 1170010], PICT [grant number 2012-0412], PICT [grant number 2012-1309], CONICET, CONICYT/FONDAP [grant number 15150012]; and the INECO Foundation.
