# Supplementary material for: Rule reactivation and capture errors in goal directed behaviour
Source: Cortex. 2018 Oct;107:180–7. doi: 10.1016/j.cortex.2017.08.027 (PMC6181800; doi:10.1016/j.cortex.2017.08.027)
Supplement: Supplementary file 1 [file mmc1.docx]

| EXPERIMENTAL PARADIGM | | | | | | | | | | | | | | | |
| --- | --- | --- | --- | --- | --- | --- | --- | --- | --- | --- | --- | --- | --- | --- | --- |
| DF |  | DB |  | NLB |  | NLB |  | DF |  | DB |  | NLO |  | NLB |  |
| 4-7 |  | 1-1 | TRIG | J-9 | TRIG | J-1 | TRIG | 1-9-8-2 |  | 1-7-7-1 | TRIG | K-C-9-2 | TRIG | F-A-7-1 | TRIG |
| 8-2 |  | 9-6 |  | 8-F |  | 3-D |  | 3-4-7-6 |  | 3-4-7-9 |  | 4-D-T-8 |  | F-5-4-Y |  |
| 2-6 |  | 4-8 |  | 6-L |  | 6-E |  | 5-1-3-5 |  | 3-6-8-3 |  | 5-R-6-F |  | G-5-6-U |  |
| 5-4 |  | 9-7 |  | 4-S |  | 8-M |  | 6-8-7-9 |  | 4-6-7-2 |  | G-7-6-T |  | 6-L-8-K |  |
| 1-7 |  | 8-8 | TRIG | X-1 | TRIG | 2-R |  | 8-1-2-4 |  | 2-5-6-8 |  | A-L-5-8 |  | R-E-8-4 | TRIG |
| 9-3 |  | 4-6 |  | 8-Y |  | H-7 | TRIG | 2-3-3-2 |  | 7-1-5-8 |  | T-H-5-9 |  | T-9-3-W |  |
| 4-4 |  | 1-8 |  | 7-Z |  | 5-i |  | 1-4-7-9 |  | 3-9-9-3 | TRIG | J-F-7-1 | TRIG | Q-G-6-7 |  |
| 3-7 |  | 8-9 |  | 1-Q |  | 8-F |  | 8-5-6-9 |  | 4-7-9-3 |  | Y-L-7-9 |  | H-7-U-5 |  |
| 1-3 |  | 4-7 |  | 3-A |  | 1-Q |  | 6-4-1-7 |  | 5-8-7-3 |  | N-8-K-4 |  | G-8-R-5 |  |
| 6-8 |  | 6-4 |  | 8-i |  | 2-P |  | 2-4-6-8 |  | 3-6-8-9 |  | D-5-F-1 |  | 5-L-T-4 |  |
| 7-4 |  | 7-7 | TRIG | E-4 | TRIG | S-4 | TRIG | 1-6-3-8 |  | 4-7-7-4 | TRIG | T-H-5-2 | TRIG | X-F-5-2 | TRIG |
| 9-2 |  | 8-1 |  | 7-T |  | 3-G |  | 2-4-7-5 |  | 2-7-8-5 |  | 2-G-6-U |  | C-5-6-Y |  |
| 3-5 |  | 5-7 |  | 2-R |  | 8-O |  | 9-4-4-9 |  | 3-5-6-8 |  | 1-H-7-R |  | C-4-5-W |  |
| 1-6 |  | 9-2 |  | 5-K |  | 4-Y |  | 5-6-7-3 |  | 2-5-7-4 |  | G-E-9-4 | TRIG | D-3-8-G |  |
| 9-9 |  | 2-6 |  | H-6 | TRIG | 9-J |  | 1-9-3-6 |  | 2-5-7-9 |  | 3-F-5-T |  | Y-J-7-6 | TRIG |
| 5-8 |  | 5-5 | TRIG | 8-O |  | N-4 | TRIG | 1-4-3-7 |  | 5-9-9-5 | TRIG | E-H-6-8 |  | 3-H-6-P |  |
| 7-2 |  | 3-4 |  | 5-N |  | 7-P |  | 4-8-3-1 |  | 3-5-8-2 |  | 5-7-R-G |  | U-3-G-7 |  |
| 1-6 |  | 2-7 |  | 9-C |  | 6-Y |  | 3-7-3-5 |  | 3-5-7-9 |  | 2-S-5-G |  | A-9-K-1 |  |
| 4-6 |  | 8-5 |  | 7-P |  | 3-A |  | 2-7-8-3 |  | 3-5-2-7 |  | E-5-G-7 |  | 7-9-R-T |  |
| 1-2 |  | 7-3 |  | 6-G |  | 7-S |  | 6-5-5-6 |  | 5-8-7-3 |  | D-4-G-7 |  | 9-F-4-T |  |
| 5-1 |  | 1-1 | TRIG | L-1 | TRIG | R-2 | TRIG | 7-8-3-5 |  | 6-4-1-9 |  | X-D-4-1 | TRIG | L-K-7-3 | TRIG |
| 4-8 |  | 1-9 |  | 5-C |  | 5-L |  | 2-7-4-2 |  | 1-3-5-7 |  | F-6-J-1 |  | P-9-i-7 |  |
| 5-7 |  | 2-8 |  | 4-D |  | 4-N |  | 6-7-8-3 |  | 3-5-7-9 |  | C-5-Y-8 |  | N-9-K-7 |  |
| 3-3 |  | 6-4 |  | 7-S |  | 8-M |  | 3-6-8-4 |  | 2-4-4-2 | TRIG | F-Z-7-4 |  | M-8-T-4 |  |
| 3-7 |  | 5-7 |  | 8-N |  | 6-P |  | 3-7-4-2 |  | 8-3-5-2 |  | 4-H-U-6 |  | D-5-6-Y |  |
| 8-2 |  | 3-6 |  | 5-J |  | 9-D |  | 7-3-3-7 |  | 4-5-7-1 |  | Q-3-J-8 |  | H-7-5-R |  |
| 1-9 |  | 9-7 |  | 6-P |  | 2-S |  | 2-8-5-3 |  | 3-5-6-8 |  | J-7-8-A |  | 8-9-J-K |  |
| 5-2 |  | 2-2 | TRIG | Q-3 | TRIG | Z-5 | TRIG | 3-9-4-6 |  | 3-5-5-3 | TRIG | U-L-7-1 | TRIG | G-A-5-3 | TRIG |
| 9-6 |  | 5-1 |  | 1-Z |  | 3-J |  | 3-4-2-6 |  | 6-1-8-7 |  | D-1-4-J |  | F-B-6-7 |  |
| 7-8 |  | 2-6 |  | 4-H |  | 7-T |  | 9-7-5-4 |  | 3-5-7-1 |  | S-6-8-A |  | D-8-4-H |  |
